# Some Criticisms Worthy of Attention

**Published:** 1920-02-07

**Authors:** 


### THE TRADE IN LUNATICS.

### Some Criticisms Worthy of Attention.

Under the above heading there appeared recently in the columns of *Truth* the plaint of a medical man, Dr. Hugh Munro, who feels strongly about the iniquities of privately-owned asylums for the insane, and about the itching palms of consultants in mental diseases. He has been quixotic (or honest) enough, so he says, to surrender the licence of one of these houses in the heyday of its prosperity, as a protest against an unjustifiable and indefensible monopoly.

We are so accustomed to *ex parte* statements which appear in the public Press regarding the enormities perpetrated in asylums, that our flesh refuses to creep over them. When, however, the criticisms emanate from one who claims inside knowledge they are worthy of more attention—if the critic's information and experience are such as to warrant us in attaching importance to his comments.

Instead of private ownership (medical, lay, or joint-stock) of licensed houses, it is suggested that they should all be placed under the ægis of the Board of Control; but the commentator in *Truth* thinks that this "would be calculated to remove one faint chance that the victim of a mistaken diagnosis has of the mistake being discovered," and suggests in turn that "management by some local authority, under the supervision and inspection of a central authority, would be best both for the patients and the public." In view of recent happenings the moment is not altogether propitious for the suggestion of doing away with private enterprise, but the State might be prepared to take this further responsibility.

### Two Points of View.

There is much to be said on this from both points of view, especially from that of the licensee. It is presumably not intended to commit the gross injustice of refraining from compensating the present owners; and it is possible that, in view of labour troubles and the increased cost of running such houses, they might welcome some scheme of expropriation. But whether the patients would be the better for the change is very doubtful. It has become an obsession with some people that all that those who have charge of the insane in licensed houses are interested in is the extraction of money from them or from their relatives, and that nothing else matters. This is simply a gross travesty of what really exists. It is becoming more and more apparent that, even putting it on the lowest basis, a place which is well run, where the patients are comfortable and happy, and where, again, the recovery-rate is good, will do a better "trade" than one which is improperly managed. And such a place will not have to go hat-in-hand to the consultant imploring his help. The accommodation, too, in licensed houses is limited, and cases must be sent *somewhere*. It is, perhaps, too much to ask the carping critics to admit that those associated with the management of licensed houses are not in all cases entirely soulless: but it is well to remember that, for the most part, the patients in these houses are in the charge of medical men who may be credited with some humanity and perchance the remnants of scientific interest!

It is this branch of the profession which has to bear the brunt time and again of abusive attacks from certain sections of the Press which ought to, and perhaps do, know better. The alliance between the mentally unstable with a grievance and the journalist in search of copy has become a time-dishonoured one.

Of course, our old friend the sane person unlawfully detained in an asylum pops his head up once more. He is the King Charles's head of the journalist. And if he is not careful he will be instrumental in altering legislation so that even dangerous lunatics, who wish to plunge razors and carving knives into editorial gizzards, will be left at large, lest one just and sane man should be wrongfully incarcerated. It is gratifying to see that even *Truth* realises that "Perhaps the possibilities of a venal conspiracy between the specialist and the asylum proprietor are too remote to call for lengthy consideration." Hints and suggestions of this kind certainly do one thing, and not the one intended. They tend to inspire a lack of confidence in the public mind so that, in some instances, necessary and urgent treatment of mentally afflicted relatives is delayed, and, in others, not only is there the worry of placing an insane relative or friend under care, but there is also an additional and unnecessary anxiety caused.

### Incarceration of Sane People?

As a matter of fact even were a sane person to find his way into an asylum it would be difficult to detain him, granted a conspiracy to do so. He has the right to see a magistrate, if he has not already seen one before being admitted; he can write to various officials, the Commissioners, the Lord Chancellor, or his visitors, Secretaries of State, and so on and his letters must be sent; he is visited at regular intervals by members of the Board of Control with whom he may have, if he so desires, a private interview. Anyone who says that the Commissioners, for example, do not fulfil their duties in this respect is simply talking nonsense and has no practical knowledge of what actually takes place. The alleged incarceration of sane people in asylums is a bogey to which the amount of attention directed is quite disproportionate.

The payment of the "unnecessary" fees to consultants is a matter which can be, and for the most part is, dealt with readily by the relatives or friends of the patients. If they choose to have the patient visited regularly by a mental specialist, who is to say them nay? Again, the estate of a person of unsound mind is carefully administered and expenditure is supervised by the Court. Needless outlays would certainly be disallowed.

